# Cutaneous and periodontal inputs to the cerebellum of the naked mole-rat (*Heterocephalus glaber*)

**DOI:** 10.3389/fnana.2013.00039

**Published:** 2013-11-18

**Authors:** Diana K. Sarko, Duncan B. Leitch, Kenneth C. Catania

**Affiliations:** ^1^Department of Anatomy, Cell Biology and Physiology, Edward Via College of Osteopathic MedicineSpartanburg, SC, USA; ^2^Department of Biological Sciences, Vanderbilt UniversityNashville, TN, USA

**Keywords:** naked mole-rat, somatosensory, tactile exploration, grasping, dentition, incisor, cerebellum, electrophysiology

## Abstract

The naked mole-rat (*Heterocephalus glaber*) is a small fossorial rodent with specialized dentition that is reflected by the large cortical area dedicated to representation of the prominent incisors. Due to naked mole-rats’ behavioral reliance on the incisors for digging and for manipulating objects, as well as their ability to move the lower incisors independently, we hypothesized that expanded somatosensory representations of the incisors would be present within the cerebellum in order to accommodate a greater degree of proprioceptive, cutaneous, and periodontal input. Multiunit electrophysiological recordings targeting the ansiform lobule were used to investigate tactile inputs from receptive fields on the entire body with a focus on the incisors. Similar to other rodents, a fractured somatotopy appeared to be present with discrete representations of the same receptive fields repeated within each folium of the cerebellum. These findings confirm the presence of somatosensory inputs to a large area of the naked mole-rat cerebellum with particularly extensive representations of the lower incisors and mystacial vibrissae. We speculate that these extensive inputs facilitate processing of tactile cues as part of a sensorimotor integration network that optimizes how sensory stimuli are acquired through active exploration and in turn adjusts motor outputs (such as independent movement of the lower incisors). These results highlight the diverse sensory specializations and corresponding brain organizational schemes that have evolved in different mammals to facilitate exploration of and interaction with their environment.

## INTRODUCTION

Although often overlooked when considering the sense of touch, information from the teeth is of critical importance to survival. Effective mastication and manipulation of food is mediated through periodontal afferent projections to sensory and motor neocortical areas as part of a sensorimotor network that integrates perception of tooth loads with fine motor control of jaw and orofacial movements (Lund, 1991; Jacobs and van Steenberghe, 1994; Turker, 2002; Trulsson, 2006; Trulsson et al., 2010). Within the neocortex of various species, representation of dentition appears to be more extensive than previously thought. Primate area 3b (primary somatosensory cortex, SI) appears to have a large dentition representation in addition to close correspondence between dental receptive fields and distinctive neuroanatomical modules within layer IV (Jain et al., 2001; Kaas et al., 2006). In fact, nearly half of the length of SI is devoted to the representation of oral structures in squirrel and owl monkeys, including dentition (Jain et al., 2001). These findings appear to extend across primate species upon examination of previous anatomical and physiological data from macaques (Dreyer et al., 1975; Krubitzer et al., 1995; Manger et al., 1996) and owl monkeys (Merzenich et al., 1978; Cusick et al., 1989).

Similar cortical modules representing dentition exist in laboratory rats (Remple et al., 2003) and naked mole-rats (*Heterocephalus glaber*; Henry et al., 2005, 2006, 2008; Henry and Catania, 2006), further indicating that columnar units of differing metabolic, connectional, and computational significance distinguish dentition across mammalian species. Naked mole-rats have a large cortical area dedicated to inputs from the dentition with over 30% of primary somatosensory cortex devoted to the two contralateral incisors alone compared to only 7% in rats (Catania and Remple, 2002; Remple et al., 2003). Within SI of the naked mole-rat, a series of modules represent the magnified upper and lower incisor representations and is readily apparent with cytochrome oxidase processing of flattened cortex sections (Henry et al., 2006). Individual modules may represent segregated inputs from distinct classes of periodontal mechanoreceptors, such as slowly adapting mechanoreceptors, rapidly adapting mechanoreceptors, or free nerve endings (thought to transduce forces applied to the rigid tooth structure without mechanical displacement of the structure; Byers, 1984; Byers and Narhi, 1999).

Naked mole-rats are fossorial, eusocial, primarily herbivorous, and functionally blind rodents. They have no pinnae and are primarily reliant on tactile and olfactory cues to navigate their subterranean environment (Hill et al., 1955; Tucker, 1981; Lacey et al., 1991; Jarvis and Sherman, 2002; Crish et al., 2003, 2006). The naked mole-rat incisors are particularly specialized, anatomically prominent, and behaviorally important, thus providing a favorable system for examining the organization of brain areas related to the dentition. Beyond the modular, expanded cortical area devoted to tactile inputs from the incisors in naked mole-rats, there is a complementary behavioral reliance on the incisors for exploration of the environment. Naked mole-rats utilize their upper and lower incisors in excavating tunnels, carrying and manipulating food items, transporting young, eating, and auto-grooming (Lacey et al., 1991). The incisors are further utilized in agonistic social interactions over competition for resources, colony defense, and competition between females when a queen is deposed. These interactions include behaviors such as: “incisor fencing” wherein two animals stand face-to-face with their mouths at right angles and incisors locked together; one pup dragging another (usually by the nape of the neck) through the tunnel system; and open-mouth gapes accompanied by hissing to threaten a conspecific (Lacey et al., 1991). Naked mole-rat incisors grow and wear rapidly, making regular tooth sharpening (performed by grinding the upper and lower incisors together) necessary. A principal diet of tubers – combined with burrowing through the fine, sandy soil characteristic of their subterranean habitat in the arid belt of the horn of Africa – also serves to wear down the naked mole-rat incisors (Lacey et al., 1991). The incisors lie exterior to the oral cavity, with the “lips” (oral folds) closing behind them (see **Figure 8**; Tucker, 1981).

Remarkably, naked mole-rats are capable of moving each individual lower incisor independently and voluntarily (Catania and Remple, 2002), an ability that might confer greater behavioral versatility in manipulating objects and interacting with their surroundings while also involving significant tactile and proprioceptive feedback. Such complex sensorimotor tasks rely on many central nervous system areas, including the cerebellum (Bower and Parsons, 2003). In contrast to the maps in the somatosensory neocortex, the cerebellum contains a patchy mosaic representation of tactile inputs from discrete body regions, or “fractured somatotopy” (Shambes et al., 1978b). This organization has been demonstrated in a wide range of species including rats (Joseph et al., 1978; Shambes et al., 1978a; Bower et al., 1981; Bower and Kassel, 1990; Bower, 2011), cats (Kassel et al., 1984), opossums (Welker and Shambes, 1985), galagos (Welker et al., 1988), guinea pigs, and mice (Bower, 1997b). Tactile projection patterns of the cerebellum vary by species with respect to the relative size, position, and topographic organization within each folium, and tend to have overrepresentations of body regions used preferentially in exploration (Bower, 2011).

Given the known behavioral and sensory importance of tactile inputs from the incisors of naked mole-rats, we hypothesized that an extensive (but fractured) somatosensory representation of the dentition would exist in the cerebellum. Based on what is known about cerebellar function in other species, presumably this representation would complement and expand upon the large neocortical area devoted to inputs from periodontal receptors, facilitating sensorimotor integration, proprioception, and cognitive processing in order to hone the acquisition of sensory information and produce effective behavioral outputs.

## MATERIALS AND METHODS

### ANIMALS

Adult naked mole-rats (*H. glaber*; *n* = 8) were maintained in a laboratory breeding colony at Vanderbilt University. Naked mole-rats were maintained in colony rooms with ambient temperatures of 30°C, 40–60% relative humidity, and free access to food (for complete housing details, see Artwohl et al., 2002). All research procedures were approved by the Institutional Animal Care and Use Committee at Vanderbilt University.

### MULTIUNIT ELECTROPHYSIOLOGICAL RECORDING PROCEDURES

A surgical plane of anesthesia was induced with an intraperitoneal (i.p.) injection of 15% urethane in distilled water (1 g/kg) in adult naked mole-rats. Additional injections of 10% ketamine (15 mg/kg, i.p.) were given as needed in order to maintain a surgical level of anesthesia. During recording each animal’s body temperature was maintained using a heating pad and hot water bottles. A midline incision was made on the head and muscle was retracted to expose the skull, with lidocaine (2%, approximately 0.5 μl) administered topically as needed. Animals were secured by a head post with dental cement and the cerebellum was exposed by craniotomy with the dura removed. The brain was protected with liquid silicon. A digital photograph of the cerebellar surface was taken for each animal and a printed copy was used to mark each recording site.

**FIGURE 1:**
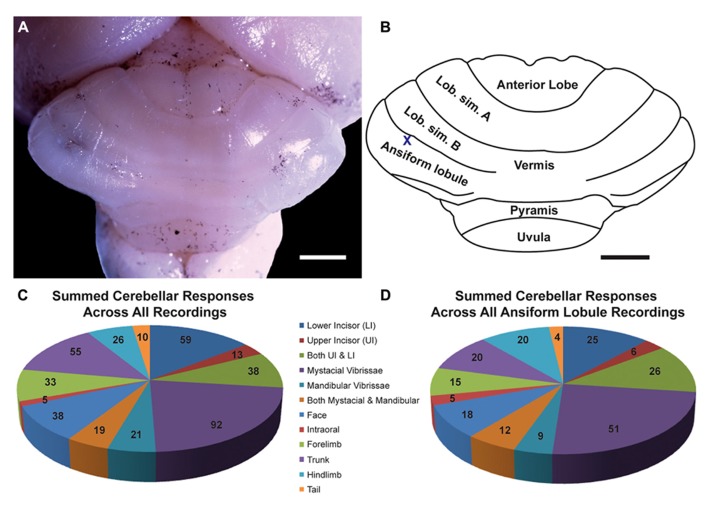
**The naked mole-rat cerebellum is shown in a dorsal view, both in a low-magnification image (A) and schematized illustration (B) to delineate the principal lobules (rostral is up; nomenclature follows; Marzban et al., 2011**). A toluidine blue dye injection within the ansiform lobule **(A)** is also represented as an X within the schematized illustration **(B)**. Data obtained from this animal is shown in **Figure 5**. Animal #030409. Scale bars: 1 mm for both **(A,B)**. **(C)** A total of 274 recording sites (summed across all folia and across all animals) yielded responses to somatosensory stimuli, the majority of which included responses to stimulation of the incisors (59 for the lower incisor alone, 13 for the upper incisor alone, and 38 for a receptive field encompassing both the upper and lower incisors) or the facial vibrissae (92 for the mystacial vibrissae alone, 21 for the mandibular vibrissae alone, and 19 for a receptive field encompassing both the mystacial and mandibular vibrissae). Responses to cutaneous stimulation of the face in addition to stimulation of the supraorbital and genal vibrissae were grouped as “face.” **(D)** Of the total recording sites from the ansiform lobule alone, a similar distribution of sensory allocation was observed with the mystacial vibrissae and incisors dominating sensory representations. Of the incisor representations, responses stimulation of the lower incisor were particularly prevalent.

Single tungsten microelectrodes (1.0 Mømega at 1 kHz) placed perpendicular to the cerebellar surface were used to record multiunit electrophysiological activity in the granule cell layer of cerebellar cortex with a particular focus on the crus II equivalent folium of the posterior lobe since this division is known to receive the majority of facial, perioral and intraoral inputs in other species (Welker, 1987). In naked mole-rats, no intercrural fissure divides the ansiform lobule into crus I and II. Crus I appears to be completely absent based on investigations utilizing zebrin II labeling (Marzban et al., 2011). Therefore, the present study targeted the ansiform lobule as the crus II equivalent in naked mole-rats, although such designation of folia is necessarily somewhat arbitrary. Each layer of the cerebellar cortex exhibits unique evoked activity properties, allowing the granule cell layer to be identified through characteristically evoked multiple unit cluster activity occurring in this layer between 25 and 400 μV above baseline noise levels (Welker, 1987). Neuronal responses were amplified and delivered to an oscilloscope and speaker. For each animal, electrode penetrations were marked on the photograph of the cerebellar surface based on vascular and anatomical landmarks. At the termination of the experiment, selected electrode penetration sites were marked with toluidine blue dye (1 g in 100 ml distilled water plus 1 g sodium tetraborate) to serve as anatomical landmarks relative to recording sites (**Figure 1**). Nomenclature identifying the principal lobules follows Marzban et al. (2011). The cerebellum was photographed using a Zeiss AxioCam HRc digital camera (Zeiss, Jena, Germany) mounted onto a Zeiss Axioskop microscope using Zeiss Axiovision 4.5 software (Carl Zeiss Microimaging, Thornwood, NY, USA) to document toluidine blue landmarks. Imported images were adjusted for brightness and contrast using Adobe Photoshop CS3 (Adobe Systems Incorporated, San Jose, CA, USA).

Receptive fields of neurons at each penetration site were mapped by stimulating the incisors, vibrissae, and body surface to establish somatotopy. Receptive fields were marked on diagrams of the naked mole-rat face and body to illustrate the region and extent of cutaneous and periodontal periphery for which stimulation elicited a response. Cutaneous stimulation of the naked mole-rat’s body with a focus on the teeth (stimulation of periodontal receptors) was performed using calibrated monofilaments (von Frey hairs). Responses to periodontal receptors of the teeth were evoked by light touch (using von Frey hairs) or light taps, as done previously in naked mole-rat (Henry et al., 2006) and primate species (Jain et al., 2001; Iyengar et al., 2007). Care was taken to ensure that each stimulus was restricted to one incisor without coincidentally producing minor movements sufficient to activate masticatory muscle proprioceptors. At each penetration site for which the receptive field included the incisors, the maximally responsive tooth was assessed first, followed by separate stimulation of the adjacent tooth in order to assess the possibility of stimulation being transmitted through tooth contacts. Additional care was taken to determine what extent of an individual incisor would generate a neural response (e.g., **Figure 3**, two responses were restricted to only the middle portion of the ipsilateral lower incisor). Penetrations were made as systematically as possible while avoiding vasculature. Following quantification of the total number of recording sites, a percentage was calculated for each major body region. A recording site was counted toward the percentage of total responses each time that site included response to stimulation of a particular body region (e.g., if the hindleg and tail each elicited a response for recording site 1, they were each counted once, and the summation of all of the responses for the tail was then divided by the total number of recording sites and multiplied by 100). Although it has been demonstrated that variability in fractured somatotopic representations exists in rats (regarding detailed spatial arrangement of body region representations in crus IIa, but not the general proportions of representations; Bower and Kassel, 1990), inter-animal variability could not be assessed in the present study due to the absence of micromapping and due to the area exposed by craniotomy varying between animals.

Recording sessions typically lasted approximately 12 h (from initial incision prior to craniotomy). After each recording session was complete, naked mole-rats were given an overdose of sodium pentobarbital (at least 120 mg/kg, i.p.) and perfused transcardially with 0.01 M phosphate-buffered saline (PBS, pH 7.2) followed by 4% paraformaldehyde (PFA) in 0.01 M PBS (pH 7.2). The brain was then removed and post-fixed in 4% PFA.

## RESULTS

Multiunit electrophysiological mapping was performed in the cerebellum of eight adult naked mole-rats, with a focus on the ansiform lobule, in order to delineate somatosensory representations. Both cutaneous and periodontal stimulation elicited responses from extensive areas of the naked mole-rat cerebellum. Somatosensory inputs were found within the anterior lobe, lobulus simplex A and B, ansiform lobule, vermis, pyramis, and uvula (**Figures 1A,B**, nomenclature follows; Marzban et al., 2011). Inputs from the incisors were found from each of these subdivisions except for the uvula. Although cutaneous responses were detected in the anterior lobe and uvula, electrophysiological sampling was too sparse to conduct further analysis.

### CEREBELLAR REPRESENTATIONS ACROSS FOLIA

A total of 274 recording sites yielded responses to somatosensory stimuli (summed across all animals, across all folia; **Figure 1C**) and an additional 81 sites were unresponsive. Of the 274 responsive sites, 110 (40%) responded to stimulation of the incisors. More sites were responsive to stimulation of the lower incisor alone (59 sites; 22% of all responsive sites) compared to the upper incisor alone (13 sites; 5%). An additional 38 sites were characterized by receptive fields that encompassed both the upper and lower incisors, rendering total lower incisor responsive sites to be 97 (or 35% of all responsive sites) and upper incisor sites to be 51 (19%).

The mystacial vibrissae were also prominently represented (**Figure 1C**), eliciting responses for 92 recording sites (33.6%). Stimulation of the mandibular vibrissae elicited responses for 21 recording sites (8%) with an additional 19 sites characterized by receptive fields that encompassed both the mystacial and mandibular vibrissae (rendering total mystacial responses to be 111, or 41%, and mandibular responses to be 40, or 15%). An additional 38 sites (14%) responded to stimulation of the face (defined here as stimulation of the supraorbital vibrissae, genal vibrissae, or cutaneous stimulation of the face) whereas 5 sites (2%) responded to stimulation of the intraoral cavity (inside of the cheek; **Figure 1C**). The trunk of the body elicited 55 responses (20%), the forelimb 33 responses (12%), the hindlimb 26 responses (9%), and the tail 10 responses (4%; **Figure 1C**).

### CEREBELLAR REPRESENTATIONS WITHIN THE ANSIFORM LOBULE

Of the total recording sites restricted to the ansiform lobule across animals (136 sites), the mystacial vibrissae and the lower incisor were also the dominant inputs (**Figure 1D**). Twenty-five sites (18%) responded to stimulation of the lower incisor alone, 6 (4%) to the upper incisor alone, and an additional 26 responded to stimulation of both (yielding total lower incisor responses of 51, or 38%, and upper incisor responses of 32, or 24%; **Figure 1D**). Stimulation of the mystacial vibrissae elicited responses from 51 of ansiform lobule recording sites (38%) whereas stimulation of mandibular vibrissae elicited responses at 9 sites (7%), with an additional 12 sites responsive to stimulation of both body regions (yielding total mystacial responses of 63, or 46%, and mandibular responses of 21, or 15%; **Figure 1D**). The face elicited 18 responses (13%), the intraoral cavity 5 responses (4%), the trunk of the body 20 responses (15%), the forelimb 15 responses (11%), the hindlimb 20 responses (15%), and the tail 4 responses (3%; **Figure 1D**).

### CHARACTERIZATION OF RECEPTIVE FIELDS, LATERALITY, AND SOMATOTOPY OF RESPONSES

**FIGURE 2:**
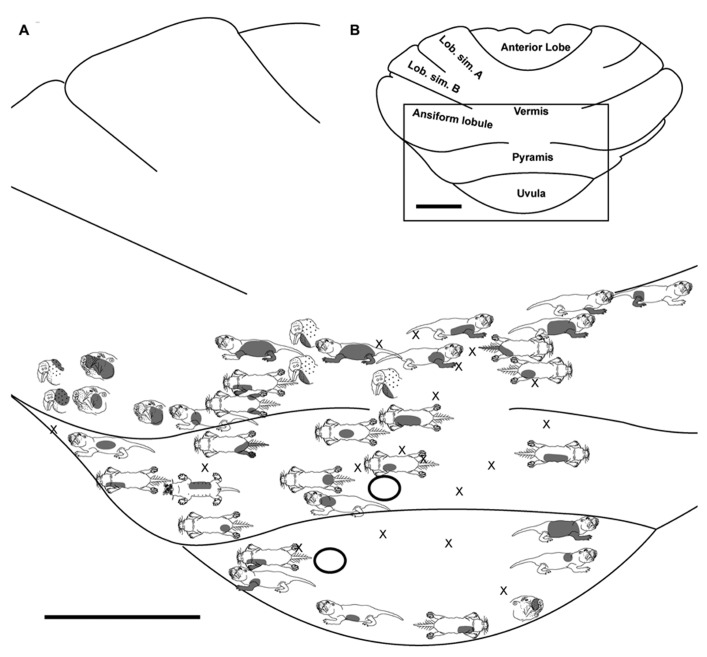
**Topography of the naked mole-rat cerebellum with electrophysiological mapping of cutaneous and periodontal responses from the ansiform lobule, vermis, pyramis and uvula. (A)** Schematic of the microelectrode-derived map of cutaneous inputs to the cerebellum labeled according to corresponding receptive fields. Dark circles outline sites injected with toluidine blue at the termination of the experiment to serve as anatomical landmarks. “X” marks designate recording sites from which no response was detected. **(B)** Illustration of the naked mole-rat cerebellum with a box delineating the area exposed by craniotomy. Dorsal view, rostral is up. Animal #031709. Scale bars: 1 mm for both **(A,B)**.

**FIGURE 3:**
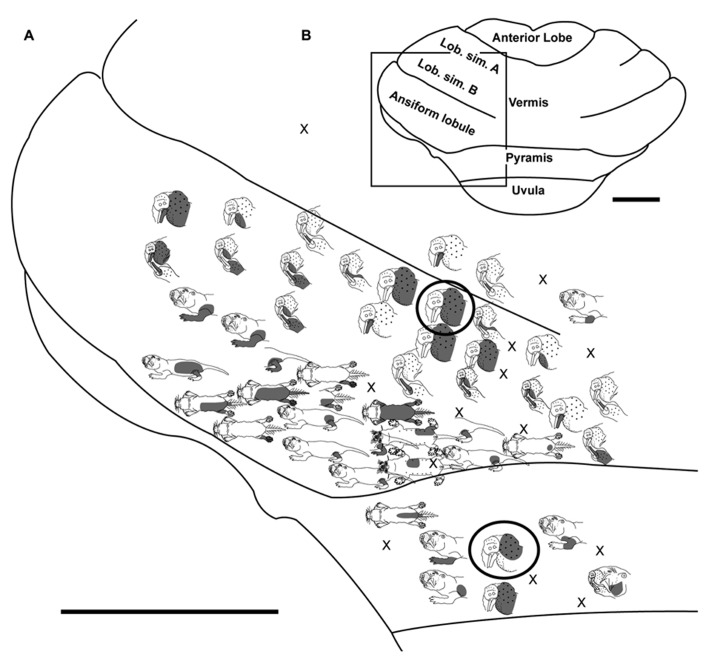
**Topography of the naked mole-rat cerebellum with electrophysiological mapping of cutaneous and periodontal responses from the lobulus simplex B, vermis, ansiform lobule, and pyramis. (A)** Schematic of the microelectrode-derived map of cutaneous inputs to the cerebellum labeled according to corresponding receptive fields. Dark circles outline recording sites injected with toluidine blue at the termination of the experiment to serve as anatomical landmarks. “X” marks designate recording sites from which no response was detected. **(B)** Illustration of the naked mole-rat cerebellum with a box delineating the area exposed by craniotomy. Dorsal view, rostral is up. Animal #121409. Scale bars: 1 mm for both **(A,B)**.

**FIGURE 4:**
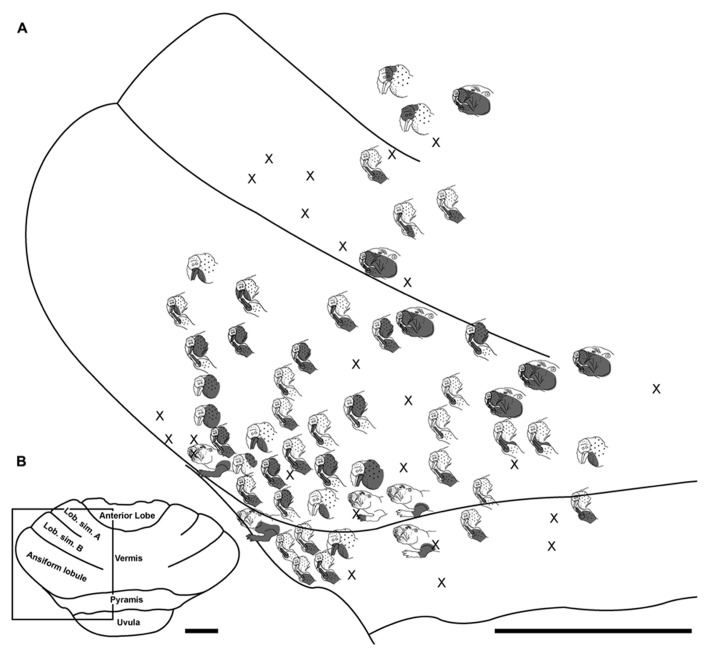
**Topography of the naked mole-rat cerebellum with electrophysiological mapping of cutaneous and periodontal responses from the lobulus simplex A and B, ansiform lobule and pyramis. (A)** Schematic of the microelectrode-derived map of cutaneous inputs to the cerebellum labeled according to corresponding receptive fields. “X” marks designate recording sites from which no response was detected. **(B)** Illustration of the naked mole-rat cerebellum with a box delineating the area exposed by craniotomy. Dorsal view, rostral is up. Animal #121509. Scale bars: 1 mm for both **(A,B)**.

**FIGURE 5:**
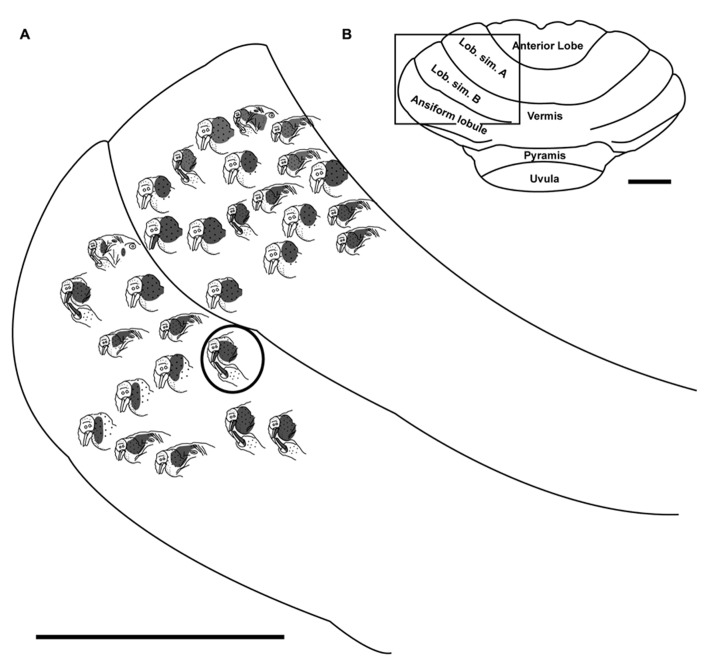
**Topography of the naked mole-rat cerebellum with electrophysiological mapping of cutaneous and periodontal responses from the lateral extent of lobulus simplex B and the ansiform lobule. (A)** Schematic of the microelectrode-derived map of cutaneous inputs to the cerebellum labeled according to corresponding receptive fields. Dark circle outlines a recording site injected with toluidine blue to serve as an anatomical landmark following termination of the experiment. **(B)** Illustration of the naked mole-rat cerebellum with a box delineating the area exposed by craniotomy. Dorsal view, rostral is up. Animal #030409. Scale bars: 1 mm for both **(A,B)**.

Receptive fields often spanned multiple body regions for a single recording site with the mystacial vibrissae and incisors frequently represented together. Other receptive fields spanned different combinations of body regions including the mandibular vibrissae and incisors (**Figure 4**); the maxillary and mandibular vibrissae as well as the incisors (**Figure 4**); the face and maxillary vibrissae (**Figure 5**); the inside of the cheek (intraoral cavity) and lower incisors (**Figure 4**); the lower incisor and lower jaw/cheek (**Figure 7**); the trunk of the body, hindlimb, and tail (**Figure 3**); and the anterior trunk extending into the neck and cheek (**Figure 2**). Although certain receptive fields spanned multiple body regions, others were restricted to a single supraorbital whisker or a portion of a single incisor (e.g., **Figures 7** and **3**, respectively, ansiform lobule).

### PYRAMIS

Within the pyramis, responses to stimulation of the incisors, face, vibrissae, forelimb, trunk of the body, hindlimb, and tail were evident (**Figures 2–4**). Bilateral responses were detected as representations of the caudal trunk and tail roughly midway between the medial and lateral extents of the pyramis (**Figure 2**) and within the lateral pyramis (**Figure 3**), but the remainder of postfacial responses were ipsilateral (**Figures 2–4**). Lower and upper incisor responses were a mix of ipsilateral and bilateral, with no apparent laterality to this distribution within the pyramis (**Figure 4**; data from two additional animals not shown). Clusters of multiple penetration sites that detected similar receptive fields were observed for the mystacial vibrissae (**Figure 3**) and lower incisor with mandibular vibrissae (**Figure 4**). Multiple, discontinuous representations of body regions were seen across animals. For instance in **Figure 3**, the forelimb is represented laterally within the pyramis followed by successive representations of the mystacial vibrissae and a repeat of the forelimb representation as one progresses medially.

### ANSIFORM LOBULE

Somatosensory-responsive regions of the ansiform lobule were extensive and exhibited a large proportion of incisor and mystacial responses, although representations of all body regions were evident (**Figures 1D** and **2–5**). Receptive fields could be quite large (e.g., bilateral receptive field spanning the forelimbs, trunk, hindlimbs, and tail) or quite restricted (**Figure 3**). Indeed, several responses were generated by stimulation to only the middle portion of the ipsilateral lower incisor rather than its entire surface area (**Figure 3**), a limited extent of the mystacial pad (although not individual vibrissae; **Figure 5**) the genal vibrissae (**Figure 7**), and the supraorbital vibrissae (**Figure 7**). Intraoral representations responsive to stimulation of the inner cheek were also localized (**Figures 3** and **4**) but were only a small proportion of the total responses (**Figures 1C,D**). Incisor receptive fields were observed to encompass the ipsilateral upper incisor alone, ipsilateral lower incisor alone, ipsilateral upper and lower incisors, bilateral upper incisors, bilateral lower incisors, and bilateral upper and lower incisors (e.g., **Figures 3** and **4**) with no apparent laterality to ipsilateral versus bilateral representations. Of the incisor responses, more ipsilateral than bilateral responses were seen for the upper incisor (30 versus 21) and lower incisor (61 versus 36). All other body region representations were predominantly ipsilateral (**Figures 2** and **4**) with a small number of bilateral postfacial representations midway between the lateral and medial aspects of the ansiform lobule. Vibrissal responses could often be detected from the same recording site as, or at adjacent recording sites to, incisor responses (**Figures 3–5** and **7**). As observed in the pyramis, multiple, discontinuous representations of body regions were seen within the ansiform lobule across animals. For instance, clusters of recording sites were observed that had similar receptive fields spanning the mystacial vibrissae, face, or forelimb (**Figure 2**); mystacial vibrissae, lower incisor with mystacial and mandibular vibrissae, forelimb, or hindlimb (**Figure 3**); face with maxillary and mandibular vibrissae as well as incisors, intraoral cavity with bilateral lower incisors, or mystacial vibrissae (**Figure 4**); and the face including mystacial vibrissae, mystacial vibrissae alone, or mystacial vibrissae with ipsilateral upper and lower incisors (**Figure 5**). These representations did not appear to have rostrocaudal continuity with the lobulus simplex or pyramis.

### LOBULUS SIMPLEX A AND B

**FIGURE 6:**
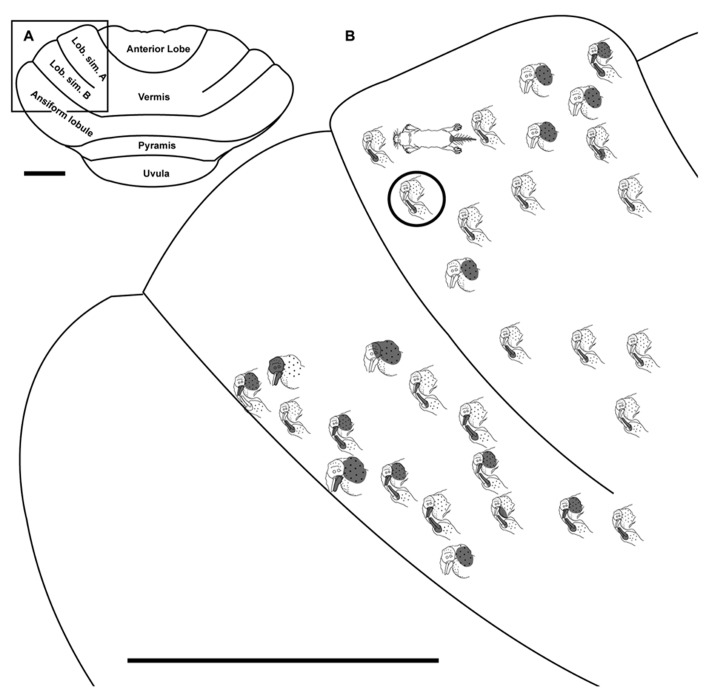
**Topography of the naked mole-rat cerebellum with electrophysiological mapping of cutaneous and periodontal responses from lobulus simplex A and B. (A)** Illustration of the naked mole-rat cerebellum with a box delineating the area exposed by craniotomy. **(B)** Schematic of the microelectrode-derived map of cutaneous inputs to the cerebellum labeled according to corresponding receptive fields. Dark circle outlines a recording site injected with toluidine blue to serve as an anatomical landmark following termination of the experiment. Dorsal view, rostral is up. Animal #050109. Scale bars: 1 mm for both **(A,B)**.

**FIGURE 7:**
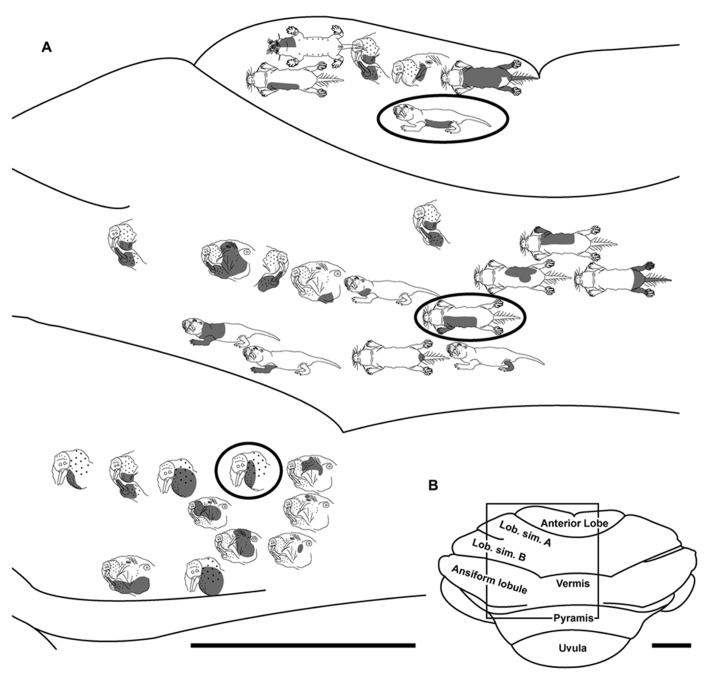
**Topography of the naked mole-rat cerebellum with electrophysiological mapping of cutaneous and periodontal responses from the anterior lobe, lobulus simplex A and B, the vermis and ansiform lobule. (A)** Schematic of the microelectrode-derived map of cutaneous inputs to the cerebellum labeled according to corresponding receptive fields. Dark circles outline recording sites injected with toluidine blue to serve as anatomical landmarks following termination of the experiment. **(B)** Illustration of the naked mole-rat cerebellum with a box delineating the area exposed by craniotomy. Dorsal view, rostral is up. Animal #110708. Scale bars: 1 mm for both **(A,B)**.

Progressing further rostrally, lobulus simplex B was primarily responsive to stimulation of the upper and lower incisors as well as the mystacial vibrissae and face (**Figures 4–7**). The mandibular vibrissae, trunk, and forelimb were represented to a lesser extent (**Figures 4–7**). As observed in the ansiform lobule, responses from the incisors and vibrissae were often detected at the same recording site or adjacent recording sites (**Figures 4–7**). Receptive fields were ipsilateral for the mystacial vibrissae, mandibular vibrissae, face, trunk, and forelimb (**Figures 4–7**) with the exception of one bilateral lower incisor and mandibular vibrissae receptive field (**Figure 7**), whereas incisor responses were a mixture of ipsilateral and bilateral responses without apparent laterality. Clusters of sites with similar receptive fields were observed for the mystacial vibrissae alone as well as the face and mystacial vibrissae (**Figure 5**) with no apparent continuity of body region representations between lobulus simplex B and A or lobulus simplex B and the ansiform lobule.

Recordings in lobulus simplex A primarily detected responses to the ipsilateral lower incisor and mystacial pad (**Figure 6**) with responses to upper incisors, face, mandibular vibrissae, and tail also present (**Figures 4** and **6**). Clusters of sites responsive to stimulation of the nose (**Figure 4**), ipsilateral lower incisor, or mystacial vibrissae (**Figure 6**) were observed. Bilateral incisor responses were again intermingled among ipsilateral responses without apparent laterality.

## DISCUSSION

### INCISOR AND VIBRISSAL REPRESENTATIONS IN THE NAKED MOLE-RAT CEREBELLUM

The present study is the first characterization of extensive representations of tactile inputs within the naked mole-rat cerebellum, particularly for the lower incisors and mystacial vibrissae. In rats, the majority of incisor inputs are located in crus IIa and b as well as lobulus simplex B, with limited representations also found in the paramedian lobule (Shambes et al., 1978a,b; Bower and Kassel, 1990). No intercrural fissure divides the ansiform lobule into crus I and II in naked mole-rats, and in fact crus I appears to be absent, leaving the ansiform lobule as the crus II equivalent (Marzban et al., 2011). Incisor representations were extensive within the ansiform lobule as well as lobulus simplex A and B of the naked mole-rat, in addition to being found in the pyramis, anterior lobe, and vermis.

Inputs from the lower incisor and mystacial vibrissae predominated throughout the naked mole-rat cerebellum (**Figure 1C**). Within the ansiform lobule (**Figure 1D**), 38% of sites were responsive to stimulation of the lower incisor and 24% of sites were responsive to stimulation of the upper incisor. By comparison, studies in rats have demonstrated 8.5% of total responses dedicated to the lower incisor and 5.3% to the upper incisor in crus IIa of the rat (Shumway et al., 1999). Further examination revealed an additional 15% of subdominant responses recorded from crus IIa following stimulation of the lower and upper incisors, yielding a total of 20.3% for the upper incisor and 23.5% for the lower incisor (Shumway et al., 2005). Thus the naked mole-rat appears to have an upper incisor representation that is approximately as extensive as that of the rat, whereas the lower incisor representation appears to be expanded.

With regard to behavioral use, the upper incisors of rats and naked mole-rats likely function in similar capacities: grasping objects and assessing the appropriate bite force for each object, which would require significant (but similar) degrees of tactile feedback. The development of independently mobile lower incisors represents a key adaptation that has allowed the naked mole-rat to manipulate and palpate objects in a subterranean environment devoid of visual cues. The mobile lower incisors thus differ physiologically and behaviorally from those of rats, which may explain the differential proportion of representation compared to upper incisors. In addition to exhibiting many sites responsive to the incisors, the naked mole-rat cerebellum was preferentially occupied by sites responsive to stimulation of the mystacial vibrissae, as shown in rats (Shambes et al., 1978b). These sites were often characterized by receptive fields that spanned both the vibrissae and incisors (e.g., **Figure 4**). Additionally, sites with receptive fields exclusive to the vibrissae were frequently adjacent to incisor receptive sites (e.g., **Figure 3**), as has been demonstrated for body regions working together to complete exploratory sensorimotor tasks (Sanes et al., 1995; Bower, 2010; van der Zwaag et al., 2013). Nearby patches may improve the cooperation between the represented body regions by forming a selectively combinatorial map of body parts that work together to perform specific behaviors (Bower, 2010) – for instance, the hand and digits in humans (Sanes et al., 1995; van der Zwaag et al., 2013). Such adjacency and potential “cooperation” between incisor and vibrissal representations may facilitate common actions involved with object manipulation and exploration in the naked mole-rat, particularly during digging and social behaviors.

### METHODOLOGICAL CAVEATS AND CONSIDERATIONS

Without comparable methodologies of detailed micromapping the 38% of responses dedicated to the lower incisor in the ansiform lobule of the naked mole-rat cannot be directly compared to the 23.5% found in crus IIa of the rat (also, beyond methodological considerations, anatomically the ansiform lobule of the naked mole-rat would not directly compare to crus IIa of the rat). However, it does seem likely that an expanded area of cerebellar cortex is dedicated to periodontal inputs, preferentially from the lower incisors, which likely reflects the sensitivity necessary for appropriate tactile and proprioceptive feedback particularly influenced by their specialized ability to move the lower incisors independently (Catania and Remple, 2002).

Receptive fields often spanned multiple body regions for a single recording site, which could be due to multiunit mapping detection of pooled neuronal activity, composed of single units with distinct receptive fields (e.g., incisor or mystacial vibrissae), or electrode placement that bordered representations of both individual body regions. However, despite the use of multiunit recording procedures, very small receptive fields were also discernible for what are likely the most sensitive body regions of the naked mole-rat: the vibrissae and lower incisors. For these body regions, receptive fields such as a group of several adjacent, ipsilateral mystacial vibrissae (**Figure 2**) or the central extent of the ipsilateral lower incisor (**Figure 3**) were evident.

A relatively large number of recording sites were unresponsive to cutaneous or periodontal stimulation (81 penetration sites across all animals; 23%) compared to rats (2%; Shumway et al., 1999). It is possible that other responses that were not tested would have been detected using a larger stimulus repertoire. Because naked mole-rats lack true “lips” (which are extensively represented in the rat cerebellum (Shambes et al., 1978a,b) and instead have oral folds that close behind the large incisors (Hill et al., 1955), it was difficult to map receptive fields pertaining to the lips, molars, tongue, and gums. It is likely that a number of these responses would be detected with further study.

### FRACTURED SOMATOTOPY

The granule cell layer of cerebellar hemispheres is known to contain a non-continuous map of the body surface, a pattern referred to as fractured somatotopy, with multiple discrete representations of the same receptive fields arranged such that adjacent body regions commonly project to spatially segregated patches within each folium (Welker, 1987; Gonzalez et al., 1993; Shumway et al., 1999). The representation of tactile inputs as a fractured somatotopy in the cerebellum of other species is thought to result from the combination of peripheral and cerebro-cerebellar tactile receptive fields (Morissette and Bower, 1996). A patchy somatotopically organized cutaneous projection pattern is distributed to the granule cell layer of the cerebellum and is present across a wide range of mammalian species including opossums (Welker and Shambes, 1985), cats (Kassel et al., 1984), rats (Shambes et al., 1978a,b; Bower et al., 1981; Woolston et al., 1981; Ruigrok and Voogd, 2000), and humans (Rijntjes et al., 1999; Bushara et al., 2001). As demonstrated in other taxa, the naked mole-rat cerebellum appeared to exhibit a fractured somatotopy with discrete representations of the same receptive fields repeated within each folium of the cerebellum (e.g., **Figure 3**, ansiform lobule, mystacial vibrissae representations), and sites with similar receptive fields clustered together (e.g., **Figure 6**, lobulus simplex A, lower ipsilateral incisor representations) adjacent to the representation of a non-continuous body surface. In crus IIa of rats, the pattern of bilateral responses and contralateral responses exhibited clear medial segregation within the fractured mosaic, which does appear to markedly differ from the pattern found in naked mole-rats. Incisor responses were frequently bilateral and were interspersed with ipsilateral responses along the full mediolateral extent of the ansiform lobule. Although fractured somatotopy was evident in naked mole-rats, an overarching pattern could not be distinguished with certainty across animals due to the use of single electrode recordings that lack the degree of “micromapping” (65 electrode penetrations/mm^2^) necessary to elucidate the detailed pattern of boundaries between body representations (Shambes et al., 1978b; Bower, 2011).

A framework of parasagittal zones activated by shared inputs has also been proposed as an alternative model to that of fractured somatotopy in cerebellar organization (Manni and Petrosini, 2004; Ruigrok, 2011; Voogd, 2011). This zonal division of olivocorticonuclear projections depicts body region representations that span all cerebellar folia in continuous rostrocaudal strips. However, because naked mole-rat body regions do not appear to be represented in parasagittal zones, the present data do not support such an organizational scheme in this species. Rather, body region representations appear to be divided into smaller, discontinuous patches with sharp transitions (rather than zonal continuity in a rostrocaudal progression), more consistent with fractured somatotopy.

### NEURAL CIRCUITRY OF DENTITION

**FIGURE 8:**
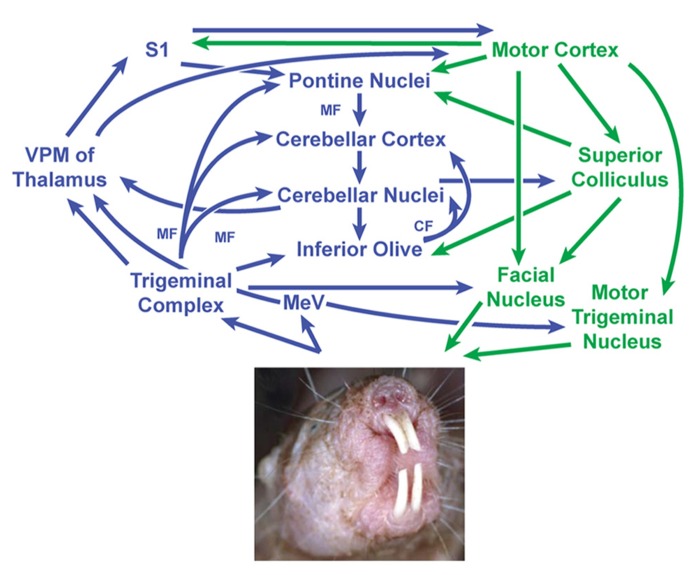
**Proposed circuit diagram for sensory inputs from the incisors (upper and lower) and motor output to the incisors (lower only), adapted from the neuronal pathways involved in sensorimotor transformations in rodent whisker movements (Bosman et al., 2010) combined with what is known about periodontal inputs in cats and ferrets (Cody et al., 1974; Linden, 1978; Gottlieb et al., 1984; Elias et al., 1987)**. The cerebellum is centrally located within this network and receives both direct and indirect projections from the trigeminal nuclei. The mesencephalic nucleus is separated from the trigeminal nuclear complex in this diagram to emphasize what are thought to be very short latency projections from periodontal receptors to the cerebellum (Elias et al., 1987) along with projections from the mesencephalic nucleus mediating jaw reflexes via the motor trigeminal nucleus. Sensory stimuli – particularly from the incisors and mystacial vibrissae – are presumably integrated to guide and optimize motor outputs involving facial motor and trigeminal motor activity (in the naked mole-rat’s case, including movements of the lower incisors). This in turn would subserve the manipulation of objects and exploration of the subterranean environment. Image of naked mole-rat reprinted with permission from Elsevier, Copyright 2006. Catania, K.C., and Henry, E.C. (2006). Touching on somatosensory specializations in mammals. *Curr Opin Neurobiol* 16, 467–473. CF, climbing fibers; MeV, mesencephalic trigeminal nucleus; MF, mossy fibers; S1, primary somatosensory cortex; VPM, ventroposterior nucleus of the thalamus (medial division).

Somatosensory inputs are known to project to the cerebellum through a number of well-established direct and indirect pathways in other species (Torvik, 1956; Clarke and Bowsher, 1962; Swenson et al., 1984; Schmahmann and Pandya, 1989; Yatim et al., 1996; Kleinfeld et al., 1999; Giannetti and Molinari, 2002; Pijpers and Ruigrok, 2006; Arenz et al., 2009), making it possible to propose a model for the neural circuitry involving cerebellar connectivity to the incisors in naked mole-rats (**Figure 8**). Periodontal mechanoreceptors in cats have been shown to project through the inferior alveolar nerve to the caudal mesencephalic nucleus (Cody et al., 1974; Linden, 1978; Gottlieb et al., 1984; Nomura and Mizuno, 1985), which in turn projects directly to cerebellar cortex in ferrets via the superior cerebellar peduncle (Taylor and Elias, 1984; Elias et al., 1987; Taylor et al., 1987). The inferior alveolar nerve also projects to each nucleus of the trigeminal complex (particularly the rostral and dorsomedial pole of the principal sensory nucleus) in cats (Marfurt, 1981) and rats (Jacquin et al., 1983). Presumably inputs from the naked mole-rat incisors would follow these pathways as well. Cutaneous input across body regions would follow both direct pathways (including mossy fiber projections from the trigeminal, cuneate, and gracile nuclei; e.g., Bermejo et al., 2003) and indirect pathways (involving the pontine and inferior olivary nuclei of the brainstem through mossy fibers and climbing fibers, respectively; for review, see Manni and Petrosini, 2004).

The cerebellum’s role is not limited to its traditionally attributed motor functions, but is also involved in a variety of sensory and cognitive tasks that may ultimately optimize the acquisition of sensory information (Gao et al., 1996; Bower, 1997a; Parsons et al., 1997; Schmahmann and Sherman, 1998; Molinari et al., 2008). The cerebellum serves as a gateway for sensorimotor integration through its central location in both sensory and motor pathways, allowing movements to be modified and optimized based on sensory feedback (Kleinfeld et al., 1999; Ito, 2000; Esakov and Pronichev, 2001; De Zeeuw and Yeo, 2005; Hoffer et al., 2005; Manzoni, 2005, 2007; Krakauer and Shadmehr, 2006; Lang et al., 2006; May, 2006; Lu et al., 2007; Middleton et al., 2008; D’Angelo and De Zeeuw, 2009; D’Angelo, 2011) through detection of errors in the sequence and timing of behaviors (Molinari et al., 2008). Within this circuitry, the inferior olive is critical in gating both self-generated and unanticipated external sensory stimuli – influenced in part by inhibitory feedback from the deep cerebellar nuclei. This affects neuronal responsiveness to peripheral stimulation (Devor, 2002) and evaluation of mismatch errors between intended and actual movement (Manni and Petrosini, 2004) which may be critical in directed movement of the naked mole-rat’s lower incisors in manipulating objects or carefully grasping pups. The cerebellum is thought to serve as an adaptive filtering mechanism that minimizes predictable, self-generated sensory inputs in favor of emphasizing those of behavioral relevance (Manni and Petrosini, 2004). This creates context-specific neural responses from peripheral cues that may be a critical component of subterranean navigation for the naked mole-rat in the absence of visual cues.

### SENSORIMOTOR INTEGRATION AND BEHAVIOR

Behaviorally, the cerebellum is instrumental in facilitating active sensory exploration, grasping, and manipulation (Hartmann, 2009), all of which are known to be functions of the naked mole-rate incisors. The majority of tactile responses in the cerebellum appear to depend on the body surfaces used principally for active sensory exploration in a given species, such that the rat has a large representation of the face (particularly the mystacial vibrissae; Shambes et al., 1978b) whereas the cat has a large representation of the forelimb (Kassel et al., 1984) and humans have a large representation of the digits (van der Zwaag et al., 2013). In naked mole-rats, a disproportionately large area of the cerebellum accommodates tactile inputs from the lower incisors, which may facilitate their role in grasping, manipulating, and exploring objects in their environment. This would include complex sensorimotor transformations related to movement of the lower incisors, a system analogous to finger-controlled grasp and palpation in humans. Human grasping involves precise manipulation of objects combined with extensive sensorimotor integration guiding the direction and degree of adequate force application (Trulsson and Johansson, 1996; Jenmalm et al., 2006; Trulsson, 2006; Milner et al., 2007; Johansson and Flanagan, 2009; Trulsson et al., 2010) similar to the sustained feedback presumably required to position the incisors (particularly with respect to self-generated movement of the lower incisors) relative to the object being explored and grasped. As such, the cerebellum would be integral in planning motor output to match environmental cues (Manto et al., 2012) – such as the size, weight, and surface friction of an object grasped by the teeth and lifted into the mouth – and in generating predictive control of grasping to counteract unexpected perturbations through grasp stability maintained by somatosensory feedback and predictive control (Ehrsson et al., 2007; Nowak et al., 2007).

Additionally, the incisors appear to be used in complementary roles of sensory exploration with the vibrissae. Use of the incisors during digging or antagonistic behaviors requires a wide gape that stretches the buccal muscles and shifts the oral folds from a horizontal to a dorsomedial stretch (Tucker, 1981; see **Figure 8**, but for the more impressive maximal gape of the naked mole-rat, see Figures 6 and 7 of Tucker, 1981). Coordinating such movements with those of the powerful masticatory muscles (which constitute 25% of total muscle mass in the naked mole-rat; Sherman et al., 1992) presumably requires extensive sensory input to fine-tune motor output in grasping food objects, tunneling, and carrying young with the incisors. This system appears to work in tandem with the facial vibrissae such that during digging, buccal evaginations close off the oral cavity and transpose the mystacial vibrissae caudally (presumably to shield the vibrissae from the mechanical stimulus onslaught involved with dirt displacement; Tucker, 1981). However, when the oral cavity is closed, the mystacial pad is situated more rostrally in a position suitable for detection and discrimination of stimuli (Tucker, 1981). The cerebellum likely plays a pivotal role in the neural networks subserving this exploration (primarily reliant on tactile hairs and the incisors) of the naked mole-rat’s subterranean environment.

## Conflict of Interest Statement

The authors declare that the research was conducted in the absence of any commercial or financial relationships that could be construed as a potential conflict of interest.
